# Designing a Case Management Mobile Health App for Violence Intervention Programs: Mixed Methods Human-Centered Design Study

**DOI:** 10.2196/79533

**Published:** 2026-02-02

**Authors:** Marianna G Salvatori, Devika Patel, Adrienne Paige Baer, Christiana Dagmar von Hippel, Jerome Wang, Daniel Goldberg, Michael Texada, Amanda Sammann

**Affiliations:** 1 The Better Lab, University of California, San Francisco, 2540 23rd Street, 4th Fl., San Francisco, CA, 94110, United States, 1 5102550453; 2 Department of Surgery, University of California, San Francisco, San Francisco, CA, United States; 3 School of Engineering, Stanford University, Stanford, CA, United States; 4 School of Public Health, University of California, Berkeley, Berkeley, CA, United States; 5 University of California San Francisco Medical Center, San Francisco, CA, United States

**Keywords:** mHealth, mobile app, violence prevention, human-centered design, RITE method, case management, mobile phone, mobile health

## Abstract

**Background:**

Hospital-based violence intervention programs have shown promise in mitigating the effects of violence, but their impact is limited by time constraints and inefficient practices faced by the violence prevention professionals (VPPs) who function as case managers. Mobile health (mHealth) apps offer the potential to enhance communication and service delivery between VPPs and clients, but few have been specifically designed for vulnerable populations.

**Objective:**

This study aims to design an mHealth app to improve communication and access to resources between survivors of violence and their VPPs using human-centered design (HCD) and iterative prototyping methods.

**Methods:**

HCD methodology was used, including rounds of Participatory Design, Low-fidelity Prototype Testing, and High-fidelity Prototype Testing. The Participatory Design phase included in-depth interviews and co-design, followed by inductive qualitative analysis to inform the mHealth app’s initial low-fidelity design. The Low-fidelity Prototype Testing phase included in-depth interviews with probing questions about the low-fidelity design, followed by inductive qualitative analysis to inform the mHealth app’s initial high-fidelity design. The High-fidelity Prototype Testing phase used the Rapid Iterative Testing and Evaluation (RITE) method and inductive qualitative analysis to rapidly collect and integrate VPP feedback into the mHealth app’s final design approved for implementation.

**Results:**

Nine VPPs participated in 3 rounds of testing and feedback. Participatory Design identified four key themes: (1) trust, (2) personal connection, (3) tailored resource curation**,** and (4) management of administrative burdens. Low-fidelity Prototype Testing identified three additional key themes: (5) intuitive and comprehensive design, (6) dynamic journey and sense of progress, and (7) standardization of verbiage and design choices. High-fidelity Prototype Testing through RITE identified 181 actionable issues, with 133 addressed, achieving a 73% impact ratio (used to measure the effectiveness of usability improvements). High-fidelity Prototype Testing identified 9 key themes, reaffirming 5 themes from prior testing sessions (themes 2, 3, 5, 6, and 7) and uncovering four novel themes: (8) control over boundaries, (9) celebration of client successes, (10) client empowerment, and (11) warm handoff. The final mHealth app version adapted from 3 low-fidelity digital representations (wireframes) to 25 high-fidelity wireframes of a mHealth app to support case management.

**Conclusions:**

The combination of HCD and RITE methodologies resulted in an mHealth app tailored to the needs of VPPs working with survivors of violence. This approach may be transferable to the development of other mHealth apps for specialized populations, although further research with larger samples would be needed to establish generalizability.

## Introduction

Violence is a public health crisis that disproportionately affects vulnerable populations [1,2]. Hospital-based violence intervention programs (HVIPs) are evidence-informed initiatives designed to address the social determinants that increase the risk of violence and to prevent repeat injury [3]. While HVIPs have demonstrated promise, their overall impact is often constrained by inefficient communication and labor-intensive case management workflows [4]. Building strong, trust-based relationships between HVIP case managers, known as violence prevention professionals (VPPs), and patients impacted by community violence who enroll in HVIPs (clients) is central to the success of these programs. However, this process is time-consuming and difficult to sustain, given limited resources and high caseloads [4].

Despite more than 350,000 violence-related emergency department visits each year in the United States [5], no mobile app, to our knowledge, currently exists to support the unique case management needs of HVIPs. Addressing community violence is critical, not only because of its immense human cost but also due to the opportunity for substantial impact on health care systems. Survivors frequently endure lasting psychological and physical trauma, including a high prevalence of posttraumatic stress disorder [6]. These burdens on individuals and families are also magnified into system-level effects. In the year of 2016, violence-related injuries cost the United States an estimated US $2.7 billion in health care expenses [7].

Previous research [8] conducted within a San Francisco HVIP identified several barriers faced by both clients and VPPs that could be mitigated through mobile health (mHealth) solutions. These challenges include the need to balance consistent emotional support with professional boundaries, offer communication that feels personal yet efficient, and recognize healing as a long-term journey requiring skill development and lifestyle changes. From this work, key design opportunities emerged: (1) maximize personal connection while regulating access, (2) enable personalized yet automated interactions, and (3) promote accountability while celebrating client progress. This research, along with other studies, suggested that a tailored mHealth app could reduce the risk of reinjury and reperpetration of violence by improving communication between clients and staff in HVIPs [5,8]. However, developing such tools requires design approaches that directly address VPP workflow challenges, including time constraints, fragmented systems, and the need to balance relationship building with professional boundaries. Translating these complex requirements into functional digital solutions demanded a systematic, user-centered methodology capable of capturing both explicit needs and latent design opportunities.

Existing digital health research has shown that mHealth apps can effectively influence health behaviors [9,10]. This potential is especially relevant for HVIPs, which serve young and vulnerable populations [11]. Given the high rates of smartphone use among emergency room patients, including those with violent injuries, mHealth apps may be a valuable tool in this context [12,13]. The recent increase of mHealth apps used to support health care delivery [9,10] highlights the importance of designing effective tools, yet many of these mHealth apps lack evidence of effectiveness and fail to involve end users in the design process [14]. Without adequate consideration of user needs, these mHealth apps risk the aforementioned ineffectiveness or potentially causing harm. This would exacerbate existing issues in the health care system [15,16]. Few digital interventions have been designed for and with vulnerable populations, and existing digital interventions for vulnerable populations often have low usage rates [17-19], indicating that they do not adequately meet user needs in the context of their daily life. This underlines an avenue for better addressing those user needs: involving users in designing mHealth apps. A human-centered design (HCD) approach offers a framework for iteratively designing and testing mHealth app concepts to enhance their acceptability and usability, which is applicable to the HVIP context.

HCD is a methodology used to design novel interventions that meet real human needs through empathetic, rigorous qualitative investigation and iterative design of prototypes [20]. HCD has been used to develop and enhance the effectiveness of mHealth tools [21] as well as other health care innovations. HCD emphasizes iteration and collaboration by incorporating user feedback into each of its 3 key stages, including the “Inspiration Phase,” “Ideation Phase,” and “Implementation Phase” [22]. The “Inspiration Phase” involves understanding the problem by thematically analyzing qualitative data gathered from key users and stakeholders through in-depth interviews in order to uncover underlying design opportunities [23]. The “Ideation Phase” entails brainstorming solutions, co-designing prototypes with users and stakeholders, and testing the desirability, feasibility, and viability of prototypes and iteratively refining them as needed [23]. The “Implementation Phase” pilot-tests refined prototypes in context, allowing for further iteration and evaluation [22]. HCD methodology therefore involves end-stage users in every step of the design process.

In health care, HCD has been used effectively to design and improve innovations that improve service and patient experience. For example, HCD was leveraged for developing a game-like simulation for lower back pain [24] and for creating virtual reality experiences to alleviate symptoms of posttraumatic stress disorder [25,26]. While many mHealth studies using HCD have focused on developing patient self-management or caregiver support tools [27-29], none have addressed mHealth apps for care teams and case management. Few mHealth design studies have documented the use of the Rapid Iterative Testing and Evaluation (RITE) method for usability testing, whereby design changes are made immediately after issues are identified (often after just 1 user). Historically, the RITE method has most commonly been used to iteratively enhance video games [30,31]. The RITE method allows for rapid iteration and continuous improvement throughout the testing process. It also adds structure to rapid testing by classifying usability issues into categories based on clarity of cause and feasibility of solution, enabling actionable issues to drive rapid changes [31]. In a mixed methods approach, HCD ensures cultural consonance, while RITE adds analytic rigor by quantifying and prioritizing usability defects in real time, a combination rarely reported in mHealth app development [32-34].

Our study aimed to apply HCD, with a focus on the “Ideation Phase,” to develop an mHealth app tailored to the needs of vulnerable populations and their care teams, with the goal of enhancing service delivery and effectiveness within HVIPs. It builds on prior research conducted during the “Inspiration Phase” [8], which laid the groundwork for this initiative. Our approach was to (1) co-design and iteratively refine the mHealth app with HVIP VPPs using an HCD and RITE mixed methods approach, and (2) report the resulting usability metrics and emerging qualitative themes for the development of future HVIP digital tools.

## Methods

### Study Design and Setting

We conducted a prospective observational mixed methods study using HCD methodology paired with the RITE method [31] to develop an mHealth app to improve communication and access to resources between clients and their VPPs.

Our study was a collaboration between the San Francisco Wraparound Project (WAP) and The Better Lab. WAP, an HVIP at Zuckerberg San Francisco General Hospital and Trauma Center, supports survivors of intentional violence by connecting them with culturally congruent VPPs at the point of care. VPPs help clients access resources, such as employment, education, legal aid, and mental health services, to reduce reinjury risk [4,23]. The Better Lab is a design research laboratory based in the University of California San Francisco (UCSF) Department of Surgery dedicated to innovating health care service solutions through HCD that enhance care and health outcomes of vulnerable populations. Research occurred from 2020 to 2024. Due to COVID-19 shelter-in-place orders, data collection took place remotely via videoconferencing in 2020‐2021. From 2022 to 2024, activities took place on-site at Zuckerberg San Francisco General Hospital.

### Ethical Considerations

This study was approved by the UCSF Institutional Review Board (protocol number 17‐23752). Verbal informed consent was received from each participant prior to their engagement in any research activities. To protect participant privacy, remote interviews (2020‐2021) were conducted via videoconferencing with participants in private locations, while in-person sessions (2022‐2024) occurred in private offices at San Francisco General Hospital. All recordings were stored on password-protected servers accessible only to the research team, and participant identifiers were removed from transcripts and replaced with codes. All data were managed in compliance with UCSF data security policies. Upon study completion, each participant received modest compensation in the form of a Visa gift card for US $50.

### Participants

Inclusion criteria for VPP participants included (1) current employment at WAP as a VPP or supervisor, and (2) active involvement in direct case management with clients enrolled in the HVIP. No minimum experience threshold was required, allowing us to capture perspectives from both new and veteran staff. Demographic data (age, gender, and race or ethnicity) were not systematically collected to preserve participant privacy in this small, identifiable sample; however, the WAP team reflects the demographic diversity of the communities they serve, with multilingual and multicultural representation among staff. Due to significant staff turnover between 2020 and 2024, a total of 9 VPPs participated in user testing, although only 4-5 were employed by WAP at any one time. Only 1 VPP, the supervisor, remained consistent throughout the entire study, participating in all 3 rounds of user testing. Participants ranged in experience from less than 1 year to more than 20 years in violence intervention work.

### Ideation Phase Methods

**Figure 1.**
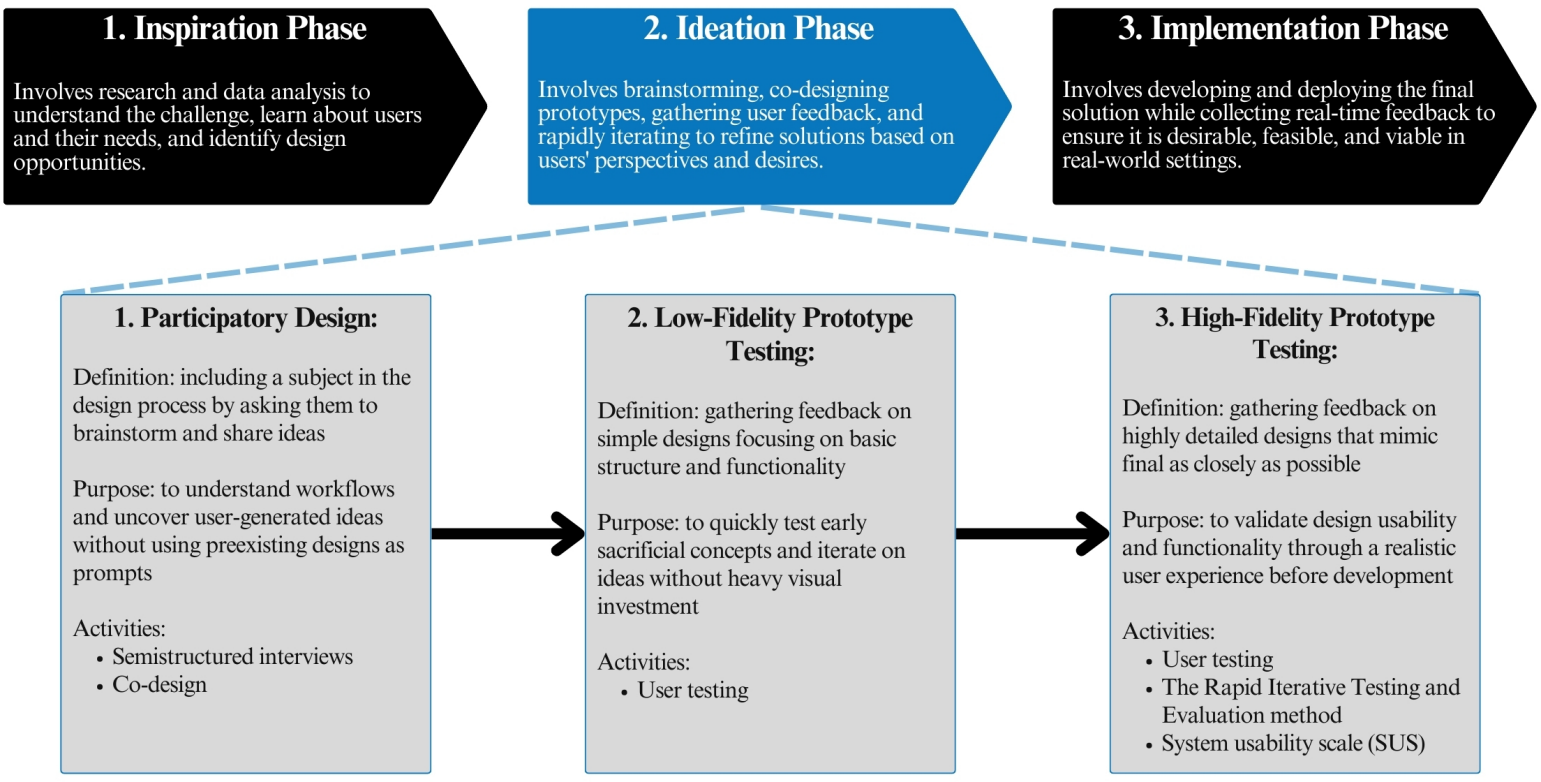
A 3-phase model of human-centered design methodology highlighting the methodologies within the Ideation Phase.

This research focused on 3 specific subphases of the HCD methodology within the “Ideation Phase”: Participatory Design, Low-fidelity Prototype Testing, and High-fidelity Prototype Testing (Figure 1). High-fidelity Prototype Testing was enhanced by the addition of the RITE method to increase the rigor of HCD with a mixed methods approach.

### Participatory Design and Thematic Analysis

Participatory Design involves the active inclusion of nondesigner stakeholders in the design process by asking them to brainstorm their own ideas. This approach aims to promote ethical engagement and mitigate traditional research power dynamics. It also aims to generate innovative concepts sourced directly from users, rather than validating pre-existing ideas [35]. Qualitative analysis of feedback from the Participatory Design phase informed the development of initial low-fidelity prototypes.

In this phase, semistructured interviews were conducted with VPPs to understand their roles, daily workflows, key client interactions, and challenges. VPPs also participated in co-design activities [36] by sketching visual concepts for the mHealth app on paper templates or guiding the interviewer in illustrating their ideas.

A remote interview lasting 60‐90 minutes was conducted in 2020 with each of the WAP’s 5 VPPs on staff at the time. A pair of researchers facilitated each session: 1 design researcher (DP) led the interview while an observer took detailed notes. Due to privacy concerns raised by the VPPs, sessions were not audio-recorded or transcribed. Instead, notes from the observing researcher were analyzed inductively using general thematic analysis [37]. Two researchers (DP and AB) independently reviewed observation notes and generated initial codes. The research team then collaboratively conducted affinity mapping [38] using the MURAL digital collaboration platform, grouping related codes into candidate themes. Key themes were identified and validated through confirmation from the VPP supervisor, who confirmed that the themes accurately reflected VPP experiences and priorities. These themes were then used to inform the design of initial low-fidelity digital representations, known as “wireframes,” of a mobile app interface.

### Low-Fidelity Prototype Testing and Thematic Analysis

The Low-fidelity Prototype Testing phase involved gathering user feedback on simple design representations focusing only on basic structure and functionality [39]. At the completion of this phase, qualitative analysis of feedback informed the development of a final low-fidelity prototype. This final low-fidelity prototype was then iterated to an initial high-fidelity prototype. This was a VPP prototype, with plans for subsequent development of the client interface.

Initial low-fidelity prototypes were created using a digital low-fidelity prototype development tool called “Balsamiq.” These prototypes included wireframes of a client dashboard, client profile, and resources section. Each wireframe included specific features identified as essential by prior research [8] and initial interview data collected during the Participatory Design phase. VPPs were presented with these prototypes and guided through a semistructured interview with prompts related to each wireframe. Feedback was used to evaluate the relevance of the low-fidelity wireframe features.

A semistructured interview was conducted and audio recorded with each of WAP’s 5 VPPs in 2020. Each session lasted 60‐90 minutes and was led by a design researcher (DP) with an observing researcher present. Two researchers (DP and AB) independently coded transcripts and then met to compare codes and resolve discrepancies through discussion. The coding team used affinity mapping in MURAL to cluster related codes and identify emergent themes. Preliminary themes were reviewed with the VPP supervisor to ensure alignment with VPP workflows and needs. Thematic analysis guided refinement of wireframes and informed the creation of a final low-fidelity prototype in Balsamiq.

At the end of the Low-fidelity Prototype Testing phase, the final low-fidelity Balsamiq wireframes were transitioned into initial high-fidelity prototypes using Figma (Figma Inc). Figma is a digital prototyping software that creates partially interactive wireframes that allow users to click through core functions, simulating realistic user experiences. Visual design choices, including layout and color, were refined using both participant feedback and established user interface design principles [40].

### High-Fidelity Prototype Testing, Thematic Analysis, and the RITE Method

High-fidelity Prototype Testing involved presenting refined wireframes of the mHealth app in Figma to simulate real user experiences [41]. These prototypes were used to test functionality, usability, and visual design before implementation. Thematic analysis and the RITE method [31] were used to guide iterative improvements and to create a final high-fidelity prototype.

The RITE method was adapted for this study as an alternative to traditional usability testing. We extended the standard 4-category RITE framework with 2 additional categories: “category 5” captured positive feedback to ensure that valued features were preserved in subsequent iterations, and “category 6” documented conflicting feedback between VPPs to guide decisions about user configurability versus standardization. This modified 6-category framework (Table 1) allowed us to not only track usability problems but also design successes and areas requiring flexible solutions.

**Table 1.** Rapid Iterative Testing and Evaluation method categories.

Category	Definition of category	Example
Classic RITE^a^ method categories		
1	Issues with an obvious cause and obvious solution that can be implemented quickly.	Text changes, relabeling buttons, and rewording dialog boxes.
2	Issues with an obvious cause and obvious solution that are difficult to implement quickly or within an appropriate time frame.	Difficult new features, current features that require substantial design and code changes.
3	Issues with no obvious cause and therefore no obvious solution.	Not enough information to assign a cause.
4	Issues due to other factors.	Test script, interaction with participant.
Added RITE method categories		
5	Positive feedback	Satisfaction, excitement, and agreement.
6	Feedback conflicts with another user’s feedback	Conflicting feedback from 2 or more users.

a RITE: Rapid Iterative Testing and Evaluation.

“Category 1” issues were fixed immediately in Figma before the next session. “Category 2” issues were either implemented between testing rounds or verbally validated with subsequent participants before committing resources. “Category 3” issues were explored through follow-up questions in future sessions or discussed with the VPP supervisor. “Category 4” issues were addressed by modifying research protocols.

“Category 5” feedback (positive responses) was documented to ensure that valued features were preserved in subsequent iterations. “Category 6” conflicts were resolved by (1) asking clarifying questions in subsequent sessions to understand underlying needs, (2) exploring design solutions offering user configurability when possible (eg, privacy toggles), or (3) making final decisions collaboratively with the VPP supervisor when configurability was not feasible.

Eight semistructured interviews were conducted: 5 with the WAP VPPs in 2020 and 4 with the WAP VPPs in 2024, with the consistent VPP supervisor participating in both rounds of interviews. Each 60‐ to 90-minute interview was led by a design researcher (AB in 2020 and MGS in 2024) with an observing researcher (DP in 2020 and DG in 2024). Participants were guided through the Figma prototypes, focusing on navigating the wireframes. They were asked to complete tasks (Table 2) and verbalize their thought process. Researchers observed difficulties and confusion, and follow-up questions probed usability, relevance of information, and emotional responses to the mHealth app.

**Table 2.** Key tasks identified for testing the mobile health app wireframes using the Rapid Iterative Testing and Evaluation method.

Wireframe titles	Key task
Sign-up	Create account with the mHealth^a^ app
Login	Log in to the mHealth app
Patients impacted by community violence who enroll in HVIPs (client) dashboard	
	Acknowledge client check-in reminder
	Filter clients by priority
	Clear client filter
	Add a client
Client profile	
	Access client profile
	Send badge to client
	Access client “Care Plan”
Care plan	
	Access client “Care Plan”
	Access client milestones
	Add a milestone
	Access client tasks and subtasks
Resources	
	Acknowledge resource updating reminder
	View employment resources
	Search for specific job
	Add job posting
	View new posting detail
	See options for interacting with job posting
	Send job posting to client
	Add job posting to correct milestone in “Care Plan”
	Search youth resources
Messaging	
	View all messages
	Navigate to new message
	Send message in an existing chat
	Add message from client as task to “Care Plan”
VPP^b^ profile	
	Access VPP profile
	Edit VPP profile
	Grant client access to other VPPs
	Access achievements

a mHealth: mobile health.

b VPP: violence prevention professional.

Each interview was transcribed, and feedback was categorized by wireframe and RITE classification. Actionable feedback (“categories 1 and 2”) was prioritized for immediate revision before the next testing session unless technical limitations prevented doing so. If a “category 1” or “category 2” change could not be implemented immediately, it was verbally tested with future participants. For example, interviewers asked questions such as: “How would you feel about including a VPP profile?” or “How would you feel if we added a photo of you?” “Category 3” and “category 4” feedback was reviewed with the VPP supervisor and subsequently addressed. To address “category 6” feedback (user disagreement), clarifying questions regarding the topic(s) were asked in future testing sessions. When disagreements persisted, solutions allowing user choice were explored. If the user choice was not feasible, final design decisions were made in collaboration with the VPP supervisor.

In addition to categorizing feedback using the RITE method, thematic analysis of data from the High-fidelity Prototype Testing phase was conducted using affinity mapping in MURAL. Two researchers (MGS and DG) independently coded interview transcripts and observation notes and then collaboratively synthesized codes into themes through iterative affinity mapping sessions. The VPP supervisor reviewed and validated final themes to ensure that they accurately represented VPP perspectives and operational realities. The emerging themes informed further improvements and inspired the creation of new wireframes. All feedback was organized by wireframe and used to guide iterative design refinements. Testing continued until saturation was reached when no novel themes emerged in the final 2 interviews. By the ninth session, feedback focused on refinements rather than new problems, indicating adequate sampling for prototype development. Together, the results from the RITE method and thematic analysis shaped the final high-fidelity prototype.

## Results

### Overview

Nineteen user-testing sessions were performed in total (5 in Participatory Design, 5 in Low-fidelity Prototype Testing, and 9 in High-fidelity Prototype Testing). A total of 11 key themes emerged from qualitative analysis of the testing sessions, with some themes emerging across several of the 3 phases (Table 3). Feedback captured during High-fidelity Prototype Testing was coded into 1 of 6 RITE categories. The mHealth wireframes were iterated toward an implementable solution, expanding from 3 initial low-fidelity wireframes to a final number of 25 distinct and interactive high-fidelity wireframes. These wireframes were approved for implementation by the VPPs and the program director of the WAP.

**Table 3.** Overview of themes by testing phase.

Theme	Description	Representative quote from VPP^a^	Phase of testing		
			Participatory design	Low-fidelity prototype testing	High-fidelity prototype testing
1. Trust	VPPs emphasized the importance of gaining trust from their clients to create space for vulnerability within the app.	“[redacted] seen me in the community but didn’t really know me. We had to build a rapport...”	✓	N/A^b^	N/A
2. Personal connection	VPPs emphasized the importance of using the app to build personal relationships with their clients.	“...because now, my client also gets a little bit of information about me.”	✓	N/A	✓
3. Tailored resource curation	VPPs expressed a need for curated, easily accessible resources within the app that are tailored to specific demographics.	"What about for the women? It’s a slight different need…finding child care, making sure the kids are in the appropriate schools...there’s been more of a lack of resources for the young women.”	✓	N/A	✓
4. Management of administrative burdens	VPPs desired to remain organized and efficient by using the app to reduce administrative burdens.	“[if I had the app] I probably wouldn’t even text. I could just send him that information to go check out that place.”	✓	N/A	N/A
5. Intuitive and comprehensive design	VPPs highlighted that the app should be intuitively designed and include only relevant features.	“If I’m just going to click on here and...apply for a job...where? What? When?”	N/A	✓	✓
6. Dynamic journey and sense of progress	VPPs wanted a way to track and manage client progress over time.	“Now, it should be like...progressing, not progressing, or completed.”	N/A	✓	✓
7. Standardization of verbiage and design choices	VPPs emphasized the need for consistent language and design throughout the app to avoid confusion.	“I think it should be standard...one of the things that I’m learning with this job, we got to speak the same language.”	N/A	✓	✓
8. Control over boundaries	VPPs expressed the need for both them and their clients to control how much or little personal information they share in the app.	“...think it should be private because...people are picky...who’s going to see this? I think the outside world should not see this.”	N/A	N/A	✓
9. Celebration of client successes	VPPs highlighted the importance of recognizing and celebrating small achievements along a client’s journey in the app.	“...he or she sees that they’re getting affirmation...it gets them a boost of confidence...everyone needs a little pat on the back.”	N/A	N/A	✓
10. Client empowerment	VPPs wanted their clients to feel empowered by having access to as much information as possible in the app to support their healing and recovery independently.	“...this is the empowerment side of the app to help the client start to follow their own care plan and do their due diligence of completing the tasks.”	N/A	N/A	✓
11. Warm handoff	VPPs emphasized the importance of directly and personally referring their clients to resources in a personal manner within the app.	“...like to warm hand off to clients, so I will contact the resource and let them know that I have a client that I’m sending over.”	N/A	N/A	✓

a VPP: violence prevention professional.

b N/A: not applicable.

### Ideation Phase Results

#### Participatory Design and Thematic Analysis Results

Participatory Design involved interviewing VPPs about their case management workflows as well as asking them to sketch concepts that could be incorporated in the mHealth app. Through general thematic analysis of these testing sessions, 4 key themes emerged: trust, personal connection, tailored resource creation, and management of administrative burdens. Trust (theme 1) was a central theme, with VPPs emphasizing the need to “build trust by following through on their word” and noting that “if clients don’t trust, they don’t share.” For instance, VPPs described scenarios where clients initially withheld concerns or personal struggles until multiple successful follow-throughs on small promises demonstrated reliability. Establishing rapport was seen as essential to successful case management. Personal connection (theme 2) was the second theme, with participants highlighting the importance of maintaining a “human factor in the app” to promote strong relationships. VPPs described wanting to share personal details such as their own music preferences or hobbies to help clients see them as real people, not just institutional representatives, which facilitated more authentic conversations about difficult topics. Tailored resource curation (theme 3) was a third theme, as VPPs described difficulty in finding and disseminating relevant information. VPPs described scenarios such as parents needing employment that accommodated childcare schedules, or a client with limited English proficiency requiring services with specific language support, situations where generic job boards or resource lists failed to meet actual needs. They noted that referring clients to a specific person, rather than a general service, was more effective, and that sorting through extensive lists could be overwhelming. Management of administrative burdens (theme 4) was the fourth theme, as participants saw potential for the mHealth app to replace administrative functions, allowing them to automate routine tasks and create more face-to-face time with clients. VPPs described spending hours each week manually updating spreadsheets, copying client information across multiple systems, and sending the same resource links repeatedly via text message. These administrative tasks reduced time available for in-person meetings and relationship building with clients.

The Participatory Design feedback and thematic qualitative analysis informed the development of an initial low-fidelity prototype in Balsamiq. This iteration included 3 wireframes (Table S1 in Multimedia Appendix 1). The wireframes incorporated specific features sketched by the VPPs, as well as themes uncovered through thematic analysis and synthesis of more nuanced feedback.

#### Low-Fidelity Prototype Testing and Thematic Analysis Results

The Low-fidelity Prototype Testing phase involved collecting feedback on the noninteractive wireframes described in Table S1 in Multimedia Appendix 1. Through general thematic analysis of these user feedback sessions, 3 themes emerged and are described below.

Intuitive and comprehensive design (theme 5) was the first theme uncovered in this phase, with participants describing how the mHealth app should be kept simple and straightforward with only necessary data and features included and clear direction for navigation. VPPs emphasized the need to balance completeness with simplicity, including essential information while avoiding cognitive overload that might discourage app use. Another theme was dynamic journey and sense of progress (theme 6), as participants described that the mHealth app should shed light on the clients’ background and experiences as well as future goals to promote effective case management that is flexible, holistic, and solution-oriented. They also highlighted the importance of tracking progress as a means for motivating the clients to continue their healing journeys. The final theme was standardization of verbiage and design choices (theme 7), meaning the mHealth app should be standardized to avoid confusion and promote inclusion. VPPs noted that inconsistent terminology created confusion when training new staff or explaining the system to clients.

The thematic qualitative analysis of feedback obtained during this phase informed the development of a final low-fidelity prototype in Balsamiq. This iteration included enhancements of the 3 initial wireframes (Table S2 in Multimedia Appendix 1). The enhancements incorporated specific feature requests from VPPs, as well as themes uncovered through thematic analysis and synthesis of more nuanced feedback.

Before transitioning to the High-fidelity Prototype Testing phase, the fidelity of the final low-fidelity wireframes (Table S2 in Multimedia Appendix 1) was enhanced from basic sketches in Balsamiq to an interactive mHealth app interface, using Figma. Qualitative interview feedback from VPPs was used to adjust and transform the final wireframes from the Low-fidelity Prototype Testing phase from 3 unique wireframes into 16 unique wireframes, adding embedded wireframes clickable within “Client Dashboard,” “Client Profile,” and “Resources” wireframes, as well as novel “Login” and “Messaging” wireframes (Table S3 in Multimedia Appendix 1).

#### High-Fidelity Prototype Testing, Thematic Analysis, and the RITE Method Results

Using the RITE method, 342 pieces of feedback were noted during High-fidelity Prototype Testing of a functional mHealth app in Figma, and each was coded into 1 of 6 categories. Between each feedback session, the 16 initial Figma wireframes were updated with adjusted features, workflows, and user interface based on user feedback. Through general thematic analysis of these testing sessions, 9 key themes emerged, which are described later in this section. The final testing results included 25 unique wireframes (Table S4 in Multimedia Appendix 1).

Examples of feedback (summaries and direct supporting quotes), RITE categories, and steps taken associated with feedback for each principal wireframe are detailed in Table 4. A total of 181 feedback items were identified as actionable and coded as either “category 1” or “category 2.” Sixty-two feedback items were designated as nonactionable and coded as either “category 3” or “category 4.” Ninety-three feedback items were positive comments and coded as “category 5.” Six feedback items were identified as disagreements between VPPs and coded as “category 6.”

**Table 4.** Feedback and coding examples from Rapid Iterative Testing and Evaluation method interviews.

Wireframe	Noted feedback or observation	RITE^a^ category assigned	Steps taken/notes
Sign-up	VPPs^b^ want the option to mark specific personal information as private during the sign-up process. Supporting quote*:* “I would like to have that option to make certain things private and certain things to the public.”	“Category 1”: Obvious cause, obvious solution, and easy to implement	Privacy toggle added for individual fields on the sign-up screen.
Login	The purpose and context of the app were unclear on the login screen. VPPs suggested adding a brief welcome or explanation. Supporting quote: “I think it’ll be important...to put ‘Welcome to Wraparound,’ let’s say, at this hospital, because people might...look at it like, well, what’s that mean?”	“Category 1”: Obvious cause, obvious solution, and easy to implement	Introductory message added to clarify that the app is part of The Wraparound Project at UCSF^c^.
Client dashboard	The filtering feature lacked visual cues, making it unclear which clients were high priority. Supporting quote: “When I clicked it, I saw that it moved around and adjusted, but as I’m perusing through [the client list] I don’t see anything that indicates to me that one is more priority than the other.”	”Category 1”: Obvious cause, obvious solution, and easy to implement	Added subtle color-coded rings to client avatars to indicate priority.
Client profile	VPPs had differing opinions on showing postdischarge timing. One wanted a clear indicator for the critical early weeks; the other felt it wasn’t needed. Supporting VPP 1 quote*:* “Those three weeks are critical, how can I help you in that critical time?” Supporting VPP 2 quote: “I wouldn’t need to know the 12 days post discharge, I don’t think that’s information on the app that I would need.”	“Category 6”: Disagreement between VPPs	Replaced “days postdischarge” with “date of injury”; discharge date included as a milestone.
Care plan	VPPs felt that tasks under milestones lacked actionable guidance, such as where or how to apply for jobs. Supporting quote: "So yeah, if I’m just going to click on here and say, apply for a job, where? What? When? How informative is this link or this check box if it doesn’t have a comment box to say what, when, how, and where? Does that make sense?”	“Category 2”: Obvious cause, obvious solution, and hard to implement	Enabled VPPs to link specific resources to individual “Care Plan” tasks (eg, job listings).
Resources	An alert prompting action appeared in the VPP interface, although the action was not possible in the prototype. Supporting quote: “So alert, administrative assistant job hasn’t been updated in over six months. Please update. So it’s giving me an alert to have to sub—fill in something?”	“Category 4”: Issue due to other factors	No action taken—issue due to Figma prototype limitations, not actual design.
Messaging	Desire for communication to feel more personal and human, rather than robotic. Supporting quote*:* “I want to interact more with the client, that would be great. I want to have that feeling of talking to a human versus talking to an app.”	“Category 3”: No obvious cause, no obvious solution	Flagged for thematic analysis—exploring ways to humanize communication.
VPP profile	VPPs appreciated that clients could view their profiles, seeing this as a way to build trust and connection. Supporting quote: “I like it a lot because now, my client also gets a little bit of information about me…and that’s empowering. That is so empowering.”	“Category 5”: Positive feedback	Positive feedback noted—reinforces current design approach and themes uncovered from thematic analysis.

a RITE: Rapid Iterative Testing and Evaluation.

b VPPs: violence prevention professionals.

c UCSF: University of California San Francisco.

Among the 181 usability issues coded as actionable (with identifiable solutions), 133 were resolved through wireframe revisions, resulting in a 73% (133/181) impact ratio. This ratio, which measures the proportion of identified issues successfully addressed during iterative testing, indicates strong effectiveness of the RITE method and is comparable with impact ratios reported in other user-centered design studies [31]. Of the 48 actionable problems that remained unresolved, many related to customizable accessibility, for example, making the mHealth app accessible in multiple languages. These did not affect core functionality for usability testing and were deemed too difficult to prototype rapidly but were noted as essential to be addressed as the mHealth app moves into the “Implementation Phase.” The “category 6” example in Table 4 illustrates our conflict resolution process: rather than choosing 1 VPP’s preference, we identified the underlying needs (tracking critical recovery period vs avoiding unnecessary detail) and designed a compromise that accommodated both through strategic placement of information.

Thematic analysis of High-Fidelity Prototype Testing identified 9 key themes, 5 of which were consistent with earlier testing phases. Participants noted the importance of personal connection (theme 2), highlighting the need for humanized interactions between VPPs and clients that foster trust and rapport. Tailored resource curation (theme 3) pointed to the need for a streamlined, individualized process to enhance clients’ access to relevant support services. Intuitive and comprehensive design (theme 5) reaffirmed participants’ desire for the mHealth app to remain straightforward and user-friendly, with only essential features and clear navigation. In addition, dynamic journey and sense of progress (theme 6) underscored the value of enabling visibility into a client’s background, present status, and future goals to support holistic, flexible case management. Standardization of verbiage and design choices (theme 7) was also emphasized to prevent confusion and promote clarity, consistency, and inclusivity.

Four additional themes emerged uniquely during this phase. Control over boundaries (theme 8) reflected the need to give both clients and VPPs greater control over who can access sensitive information, helping to protect psychological safety and respect privacy. VPPs described situations where clients might be comfortable sharing their recovery progress with their assigned caseworker but not want other staff members viewing person details. Similarly, VPPs expressed varying comfort levels sharing personal information such as their own photographs or biographical details. Celebration of client successes (theme 9) highlighted the motivational power of recognizing and affirming clients’ progress. VPPs wanted to acknowledge milestones such as a client’s first week of consistent employment, completion of academic course, or attending their first therapy session. Client empowerment (theme 10) spoke to the importance of supporting clients as active participants in their healing journey, with tools that encourage autonomy and ownership. Rather than VPPs controlling all aspects of the care plan, clients could view their own milestones, mark tasks as complete, and access resources independently, shifting from a model where clients passively received services to one where they actively drove their recovery. These 3 themes had also surfaced during the earlier Inspiration Phase of this work [8]. Finally, warm hand-off (theme 11) highlighted the importance of humanizing referrals by ensuring that clients are personally connected to external resources, rather than being left to navigate systems on their own. For example, rather than simply texting a client a link to a job-training program, VPPs described calling the program first, speaking with a specific contact person, confirming eligibility and availability, and then introducing the client by name, creating accountability on both sides and significantly increasing the likelihood of successful connection.

**Figure 2.**
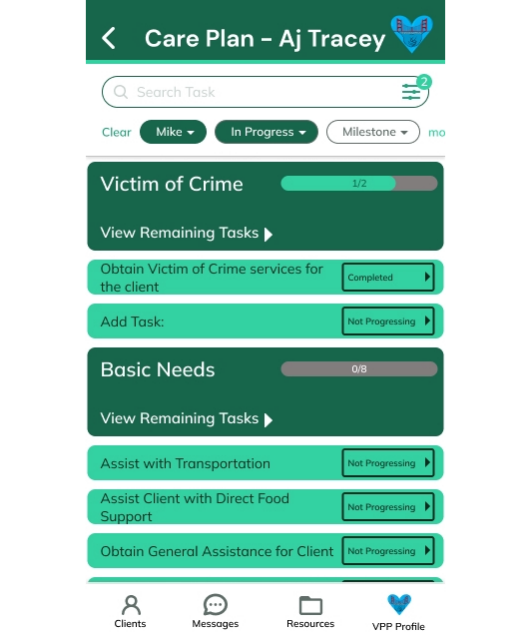
High-fidelity WrapApp client care plan wireframe. VPP: violence prevention professional.

**Figure 3.**
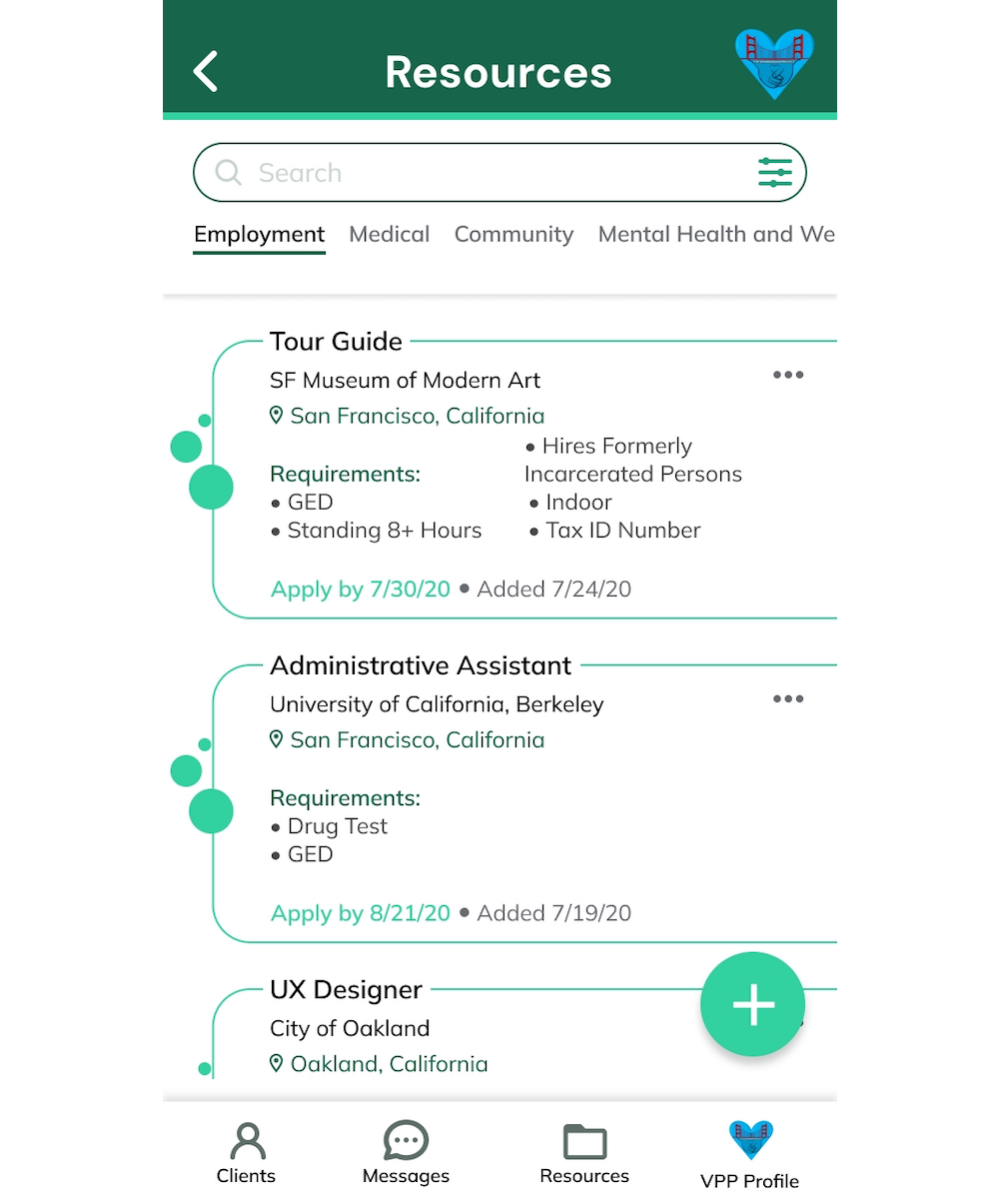
High-fidelity WrapApp resources library wireframe. GED: general educational diploma; SF: San Francisco; UX: user experience; VPP: violence prevention professional.

**Figure 4.**
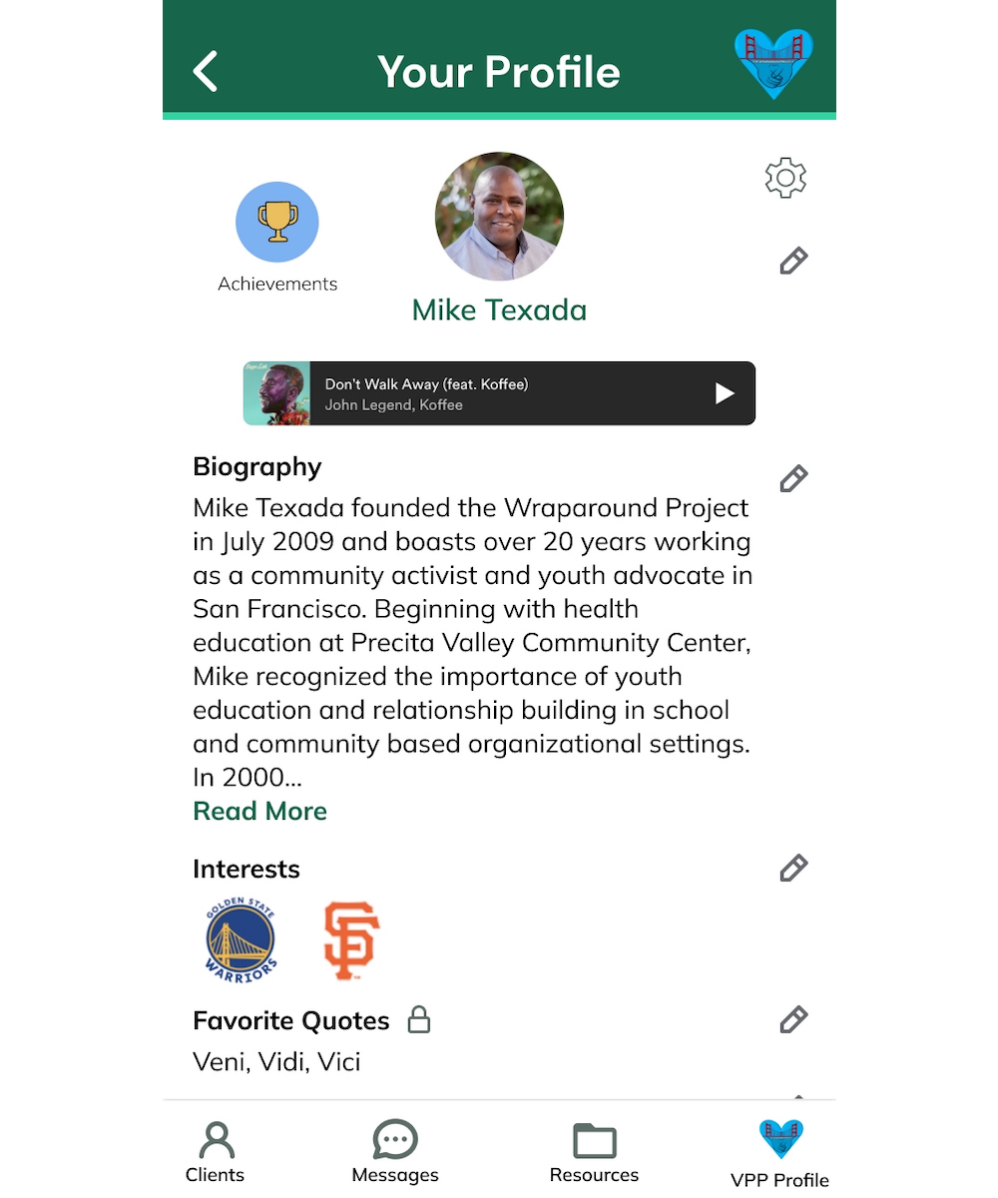
High-fidelity WrapApp violence prevention professional caseworker profile wireframe. VPP: violence prevention professional.

These findings led to several feature enhancements. Specifically, a customizable “Care Plan” was added that uploads as a standard template of general milestones, tasks, and subtasks for HVIP clients designed from experienced VPP input but can be tailored to individual needs. The “Care Plan” wireframe shown in Figure 2 empowers clients to personalize their healing journey and manage their goals and timelines. To minimize overwhelm and enhance relevance, the ability to link specific resources from the “Resource” wireframe shown in Figure 3 directly to relevant tasks within the “Care Plan” was integrated. The “VPP Profile” wireframe shown in Figure 4 was also added to enhance the human touch element of the mHealth app, as participants noted that it was important to share personal information about themselves to build trust with clients. However, recognizing the varying comfort levels around information sharing, personalized privacy settings were added to allow VPPs to customize the sensitive information displayed on their profiles. An out-of-office automation feature was also implemented to prevent VPPs from being contacted outside working hours while providing emergency contact options for clients. Finally, to facilitate the “warm handoff” approach, features enabling VPPs to connect clients with resources through direct contacts were incorporated, ensuring a more guided and meaningful support system. More examples of the 25 final high-fidelity wireframes and associated features are shown in Table S4 in Multimedia Appendix 1*.*

## Discussion

### Study Overview and Key Findings

We integrated HCD with rapid iterative testing to develop a prototype mHealth app tailored to the workflow needs of an HVIP. Across 9 rapid-cycle testing sessions, 133 of 181 identified actionable usability issues were addressed, yielding an impact ratio of 73%. The prototype expanded from 3 initial wireframes to 25 high-fidelity screens. This process clarified 3 design priorities for VPPs.

First, features needed to strengthen trust while preserving professional boundaries, which informed inclusion of profile photographs, recognition badges, and out-of-office automation. Second, a client-owned care plan with editable milestones and visible progress indicators supported motivation and accountability. Third, personalized resource sharing mattered more than static lists, leading to workflows that allow a VPP to send a resource directly into a client’s care plan or message thread and include a named point of contact when available. Together, these results illustrate how combining HCD with RITE can translate complex case management relationship-based requirements into concrete interface designs for a highly individualized, functional, and desirable mHealth tool that is useful for partner organizations and attuned to the complex needs of their users.

### Comparison With Existing Research

This subsection explains how our approach aligns with and extends prior guidance on sustainable digital health development, with particular attention to end user engagement and equity. As mHealth technologies play a central role in making continuous health care possible [42], growing evidence shows that mHealth projects succeed when end users shape every design phase. Consistent with An et al [43], we engaged end users throughout design to support relevance, usability, and longer-term adoption readiness. In parallel with World Health Organization guidance on digital interventions, we prioritized deep user engagement and attention to equity considerations during design, given the vulnerabilities and barriers faced by violence survivors and the caseworkers who support them [44]. Our contribution extends this literature in 3 ways. First, the primary end users were a specialized workforce delivering relationship-based, trauma-informed services, rather than patients engaging in self-management. Second, we paired participatory design and qualitative thematic analysis with rapid-cycle usability testing that generated quantifiable, actionable defect tracking during prototyping. Third, the design targets a dual-user ecosystem, where staff workflows shape what is feasible and safe for eventual client-facing functions.

Many existing mHealth apps are built for large, relatively uniform user populations and emphasize generic self-management activities such as tracking diet or medication adherence [45-47]. By contrast, our prototype was developed for a highly specialized care context in which relationships, trust, emotional safety, and boundary setting are foundational to effective use. This study demonstrates a replicable approach to designing digital tools that accommodate diverse workflows and emotionally sensitive communication within HVIPs, addressing a gap in documented iterative development processes for dual-user health care systems.

### Design and Clinical Implications

Our mHealth app has the potential to enhance the delivery of HVIP services. Operationally, the prototype consolidates tools such as spreadsheets, paper notes, and ad hoc texting into a single dashboard, with the goal of reducing administrative burden and preserving time for client engagement. Features such as the VPP Profile and Client Profile**,** in-app messaging, and achievement celebrations were designed to foster trust, enhance communication, and strengthen the therapeutic alliance between clients and VPPs. In addition, the app’s ability to provide clients with a tangible view of their healing progress through the “Care Plan” feature can boost motivation, increase self-efficacy, and promote engagement. Client empowerment was operationalized as making the plan visible, editable, and progress-oriented, so clients can see what they are working toward, what steps are next, and what has already been completed. In case management practice, this can support motivation between visits and reduce repeated explanation during time-limited contacts. VPPs can also use the app to provide real-time resource referrals with warm handoffs, ensuring better continuity of care and improved client outcomes. In practice, a warm handoff involves more than sending a link. Caseworkers often contact an organization first, identify a named person, confirm eligibility requirements, and then introduce the client to that contact. The prototype supports this workflow by allowing caseworkers to attach a resource to a specific care plan task, include application instructions, and share a named contact when available, reducing the likelihood that clients are left to navigate complex systems alone. Finally, the app promotes clearer boundary setting by offering structured communication channels and time-sensitive prompts that help both parties maintain role clarity.

### Methodological Contributions

This subsection highlights how pairing HCD with rapid iterative testing can increase speed and rigor during prototype refinement for complex interventions. Our approach of combining this modified RITE method with HCD provides a scalable and agile framework for mHealth app development, particularly in complex, high-stakes environments where deep user engagement and system integration are essential. Traditional usability testing in HCD involves limited rounds of user feedback and freezes a prototype until an entire round of feedback is complete [48]. In contrast, RITE facilitates immediate design modifications, making the development process more agile and responsive [31]. By integrating HCD with rapid iterative testing, we continuously incorporated caseworker feedback during prototyping, supporting a responsive development cycle aligned with the needs of HVIPs. Although rapid-cycle iteration can be resource intensive, the approach is adaptable, and teams can calibrate how frequently changes are implemented between sessions based on available design and development capacity. For example, RITE changes do not necessarily need to be comprehensively implemented between participants. This option would allow RITE to be approachable for organizations that may be low on time or resources. We ourselves have demonstrated in this paper an approach to modifying RITE to fit our own organizational needs.

We also expanded the classic 4 RITE codes by adding categories for positive feedback and for conflicting feedback. Capturing what users were most positive about ensured that later revisions did not inadvertently remove valued elements and allowed us to continue incorporating features that were favorable. Documenting conflicts between VPPs helped the team decide when a configurable option, rather than a single global solution, was warranted. This allowed us to maintain consistency while accommodating variability in case management approaches. The enhancements to the RITE method can be adapted to suit various community-based interventions and programs that require rapid iteration and responsiveness to user needs, particularly in multidisciplinary settings where individualized interventions are critical, such as community mental health work and oncology patient navigation.

### Limitations and Future Research

Several limitations should be considered. The sample was small (9 caseworkers) and drawn from a single HVIP, which limits generalizability. In addition, this phase focused on the caseworker-facing prototype; client input is still needed before finalizing a client-facing experience. Staff turnover also limited longitudinal participation across phases. Future studies should explore the applicability of this methodology to larger, more diverse user groups and settings.

Next, we will design and test the client-side interface using the same iterative methods and then pilot the combined system with WAP caseworkers and clients. Looking ahead, integration with an electronic health record such as Epic could enhance coordination between violence intervention teams and clinical providers. Since 2023, caseworkers in the WAP have documented client progress in Epic, which increases visibility of nonmedical needs for clinical teams. However, clients typically do not have a clear, usable view of the recovery plan described in clinical documentation. From a technical standpoint, integration could be approached by mapping selected care plan fields to structured data elements and exchanging data through Epic-supported interfaces (eg, standards-based application programming interfaces where available). Implementation would require clear governance decisions about what information is appropriate for bidirectional exchange, role-based access controls for sensitive social and safety data, and auditability of changes made by staff versus clients. Additional challenges include aligning terminologies across systems, minimizing duplicate documentation burden for caseworkers, managing consent and privacy preferences for trauma-related information, and ensuring usability within existing workflows. These considerations will inform our implementation-phase pilot planning. This pilot will include surveys, usage metrics, and qualitative feedback and will also assess workflow fit alongside existing documentation practices in Epic.

### Conclusions

A mixed methods approach to mHealth app development that combines RITE testing and HCD methodology strengthens the iterative design process, particularly for vulnerable populations. The detailed user feedback and agile design allowed us to rapidly adapt the app to the needs of VPPs, ensuring its usability and acceptability for VPPs. This approach is a promising way to develop customized, culturally congruent digital tools for social care programs and, if validated in larger trials, could serve as a blueprint for other violence prevention and community health interventions.

## Supplementary material

10.2196/79533 Multimedia Appendix 1 Tables of WrapApp wireframes in development from low to high fidelity.

## Abbreviations

HCD: human-centered design
HVIP: hospital-based violence intervention program
RITE: Rapid Iterative Testing and Evaluation
UCSF: University of California San Francisco
VPP: violence prevention professional
WAP: Wraparound Project
