# Supplementary material for: Designing a Case Management Mobile Health App for Violence Intervention Programs: Mixed Methods Human-Centered Design Study
Source: JMIR Form Res. 2026 Feb 2;10:e79533. doi: 10.2196/79533 (PMC12863242; doi:10.2196/79533)
Supplement: Multimedia Appendix 1 [file formative-v10-e79533-s001.pdf]

Multimedia Appendix 1. WrapApp Wireframe Tables

Table S1. Initial low-fidelity wireframes, associated themes from Participatory Design, associated wireframe actions and features, and detailed wireframe images

Wireframe Titles	Themes	Associated Actions and Features	Wireframe Example
Client Dashboard	● Trust<br>● Management of administrative burdens	*Action*:<br>● Scroll through list of clients for consistent caseload tracking, emphasizing check-in status to continue building trust with clients<br><br>*Features*:<br>● Client avatar, client contact information, client 'last check-in', client 'upcoming events', client 'assignments'	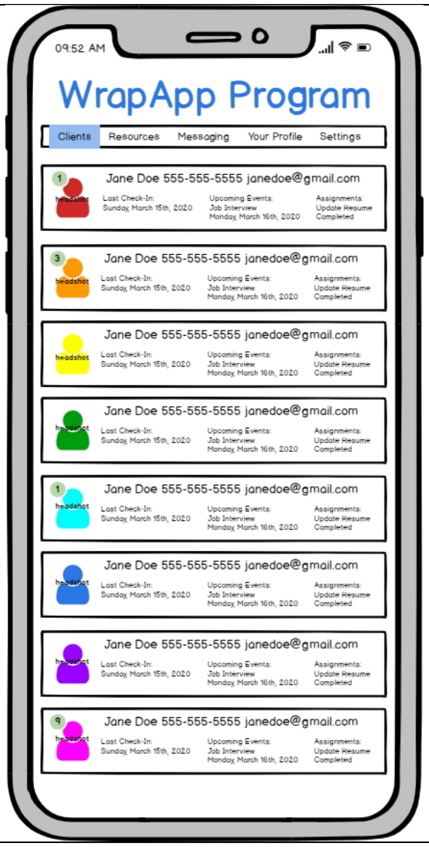

<table>
<tr><td></td><td></td><td></td><td></td></tr>
<tr>
<td>Client Profile</td>
<td>● Personal Connection</td>
<td>Action:
● Review details for a specific client that describe client’s background

Features:
● Client contact information, client avatar, client ‘additional notes’, client ‘current tasks’, client ‘upcoming tasks’</td>
<td>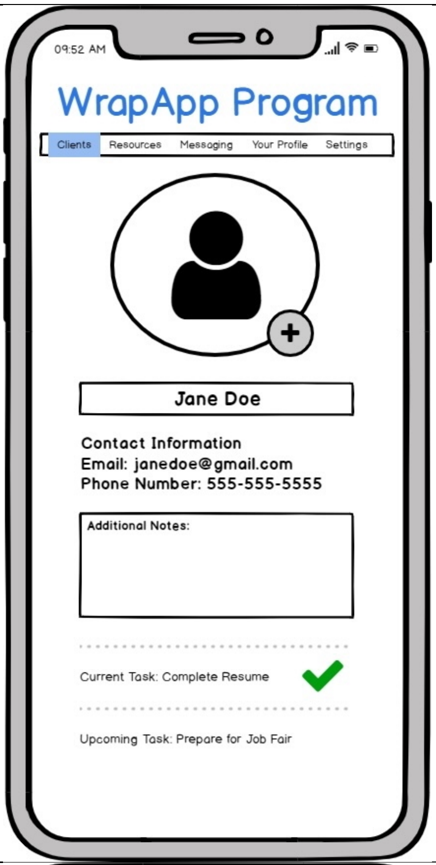
</td>
</tr>
<tr>
<td>Resources</td>
<td>● Tailored resource curation</td>
<td>Action:
● Review and scroll through job listings that are approved by the Violence Prevention Professionals (VPPs)

Features:
● Name of organization, jobs available within organization, employer contact info, application deadlines, date of last verification, ability to edit resources</td>
<td>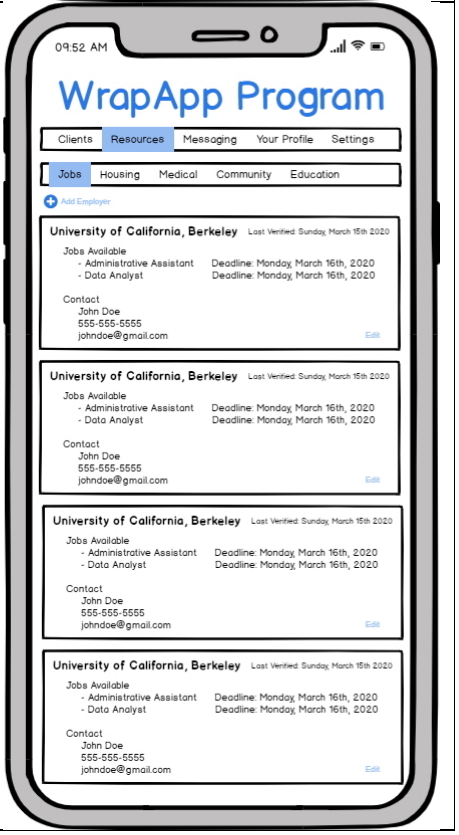
</td>
</tr>
</table>

Table S2. Final low-fidelity wireframes, associated themes from Low-fidelity Prototype Testing, associated wireframe actions and features, and detailed wireframe images

Wireframe Titles	Themes	Associated Actions and Features	Wireframe Example
Client Dashboard	● Intuitive and comprehensive design<br>● Standardization of verbiage	**Action:**<br>● Scroll through list of clients with most relevant information displayed in an organized and standard manner for consistent caseload tracking, emphasizing check-in status to continue building trust with clients<br><br>**Features:**<br>● Client name(s), client avatar, client contact information, client 'last check-in', client 'upcoming events', client 'needs', filter for clients based on 'priority' and 'status'	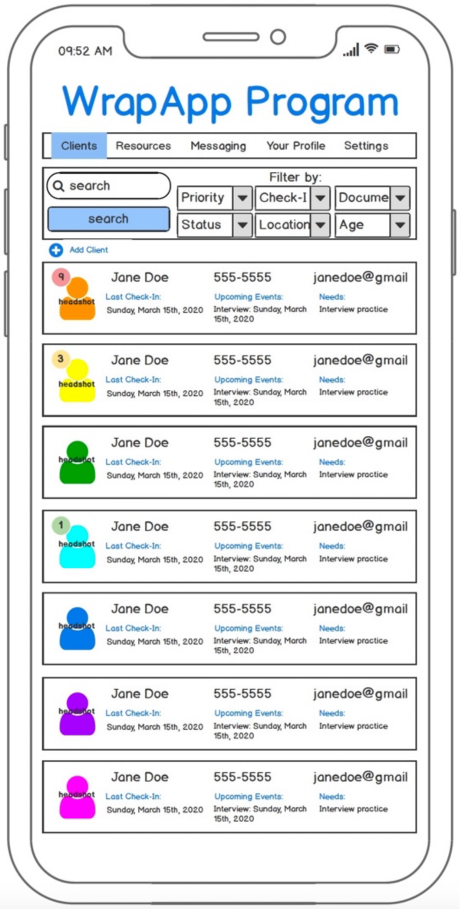

Client Profile	● Dynamic journey and sense of progress	**Action:**<br>● Review details for a specific client that describe client's background and includes milestones that promote a quick return to life post-injury<br><br>**Features:**<br>● Client name, client contact information, client avatar, client 'additional notes', client current and past 'tasks', client notifications, client 'status', client 'priority', client milestone timeline	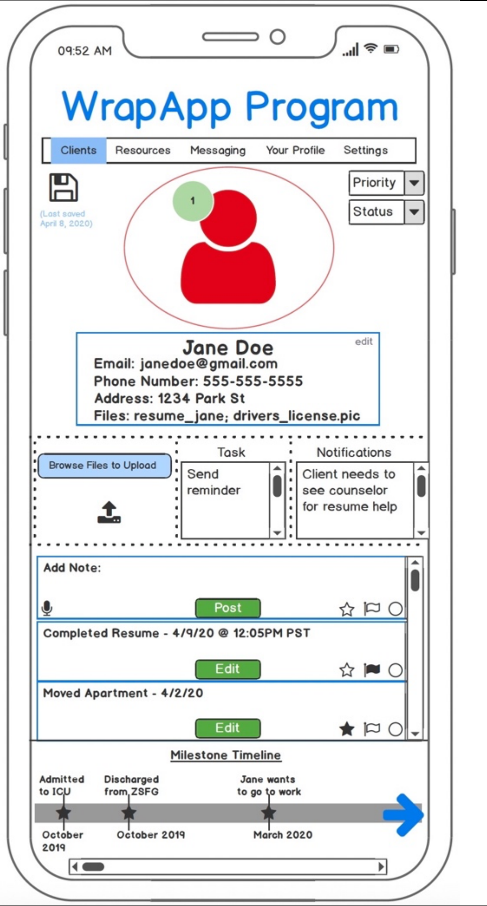

Resources	● Standardization of verbiage and design choices	**Action:**<br>● Review and scroll through standardized display of job listings that are approved by VPPs<br><br>**Features:**<br>● Name of organization, jobs available within organization, employer contact info, application deadlines, date of last verification, job requirements, ability to edit resources, ability to send resource to clients, filter for jobs	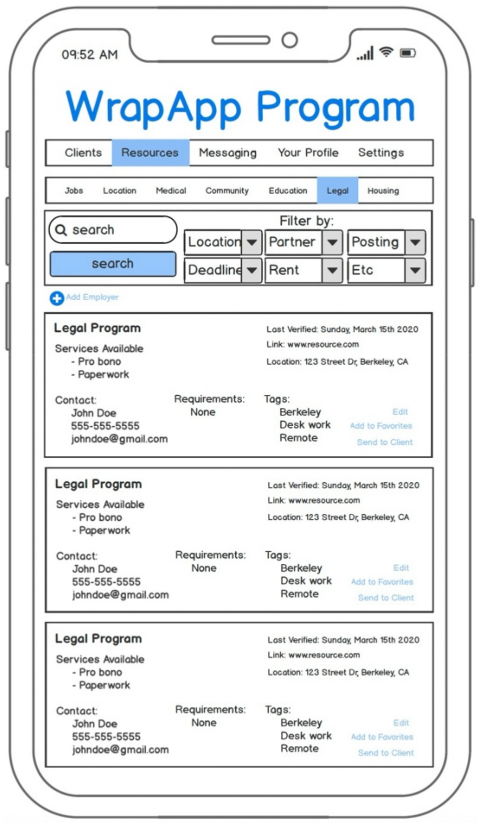

Table S3. Initial high-fidelity wireframes, associated themes from Participatory Design and Low-fidelity Prototype Testing, associated wireframe actions and features, and detailed wireframe images

**Wireframe Titles**	**Themes**	**Associated Actions and Features**	**Wireframe Example**
Sign-up	*N/A*	*N/A*	*None*
Login	● Trust<br>● Standardization of verbiage and design choices<br>● Intuitive and comprehensive design	**Action:**<br>● Log-in to the mobile health (mHealth) application (app) through a secure portal that is welcoming, simple, and easy to use<br><br>**Features:**<br>● Welcome message, mHealth app logo, login fields, option to create an account, option to reset password	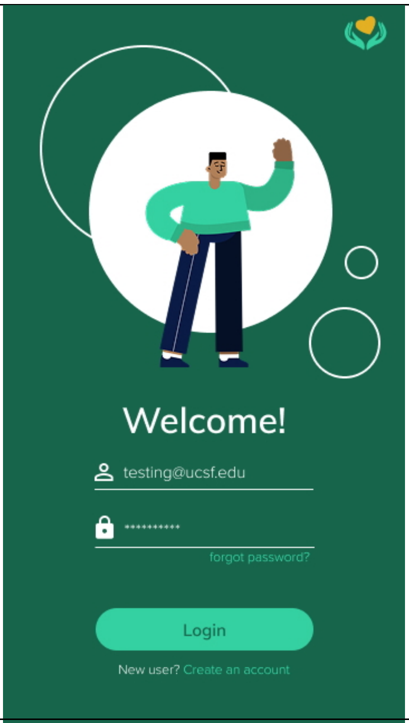
Client Dashboard	● Intuitive and comprehensive design<br>● Standardization of verbiage<br>● Trust<br>● Management of administrative burdens	**Action:**<br>● Scroll through list of clients with most relevant information displayed in an organized and standard manner for consistent caseload tracking, emphasizing check-in status to continue building trust with clients<br><br>**Features:**<br>● Client name, client avatar, client contact information, client 'last check-in', client 'upcoming events', client 'needs', filter for clients, search bar,	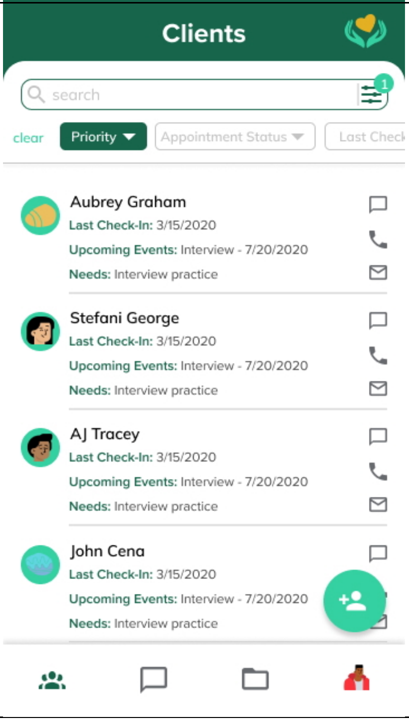

		quick actions (call, text, email)	
Client Profile	● Dynamic journey and sense of progress<br>● Personal connection	**Action:**<br>● Review details for a specific client that describe client's background and includes milestones that promote a quick return to life post-injury<br><br>**Features:**<br>● Client name, client avatar, client contact information, client 'priority', client hospitalization status, client 'tags', client 'tasks', client 'notes', quick actions (message)	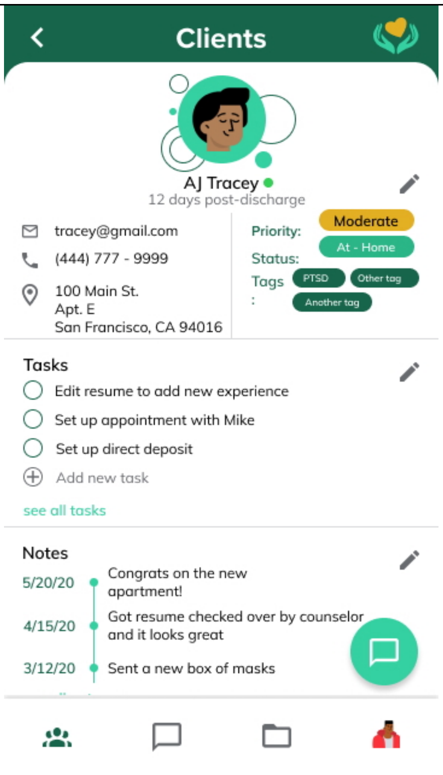
Care Plan	*N/A*	*N/A*	*None*
Resources	● Tailored resource curation<br>●Standardization of verbiage and design choices	**Action:**<br>● Review and scroll through standardized display of job listings that are approved by the Violence Prevention Professionals (VPPs)<br><br>**Features:**<br>● Name of organization, application deadlines, date job was added, job requirements, ability to edit resources, ability to send resource to clients, filter for jobs, search for jobs	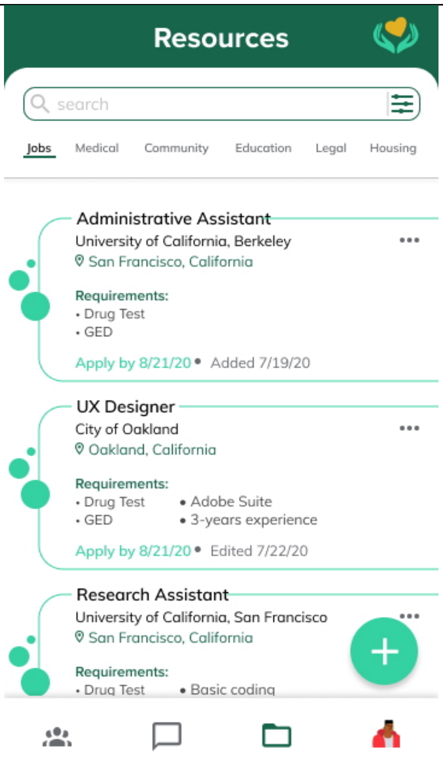

Messaging	● Personal connection<br>● Trust	**Action:**<br>● Chat or call client through secure messaging feature<br><br>**Features:**<br>● Recipient field, send text option, send voice memo option, send resource option	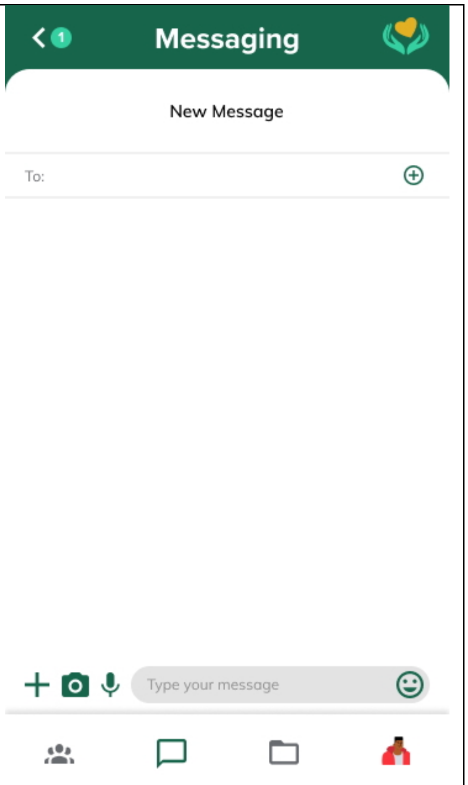
VPP Profile	*N/A*	*N/A*	*None*

Table S4. Final high-fidelity wireframes, associated themes from High-fidelity Prototype Testing, associated wireframe actions and features, and detailed wireframe images

**Wireframe Title**	**Themes**	**Associated Actions and Features**	**Wireframe Example**
Sign-up	● Control over boundaries<br>● Intuitive and comprehensive design<br>● Standardization of verbiage and design choices	**Action:**<br>●Create a Violence Prevention Professional (VPP) account for the mobile health (mHealth) application (app) with adjustable privacy settings on standardized and minimal input fields<br><br>**Features:**<br>● VPP Name, VPP date of birth, VPP contact information, VPP spoken languages, VPP password, VPP biography, VPP interests, VPP favorite song, VPP favorite quote	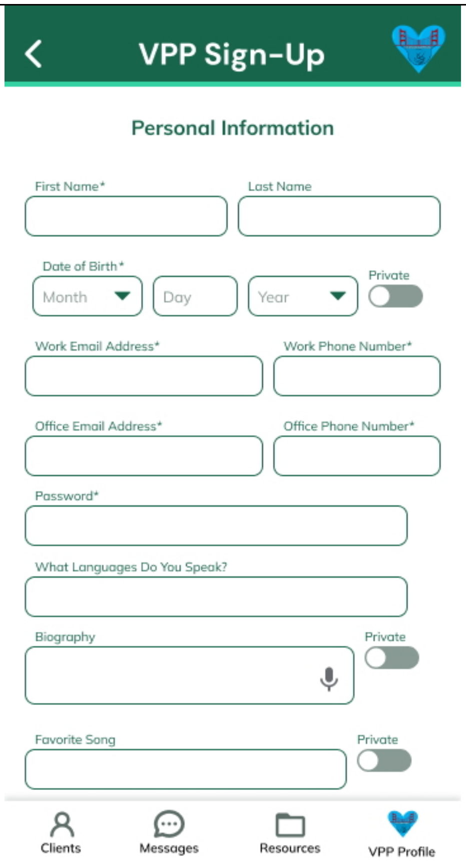

Login	● Standardization of verbiage and design choices<br>● Intuitive and comprehensive design	**Action:**<br>● Log-in to the mHealth app through a secure portal that is welcoming, simple, and standardized<br><br>**Features:**<br>● Welcome message, WAP logo, login fields, option to create an account, option to reset password	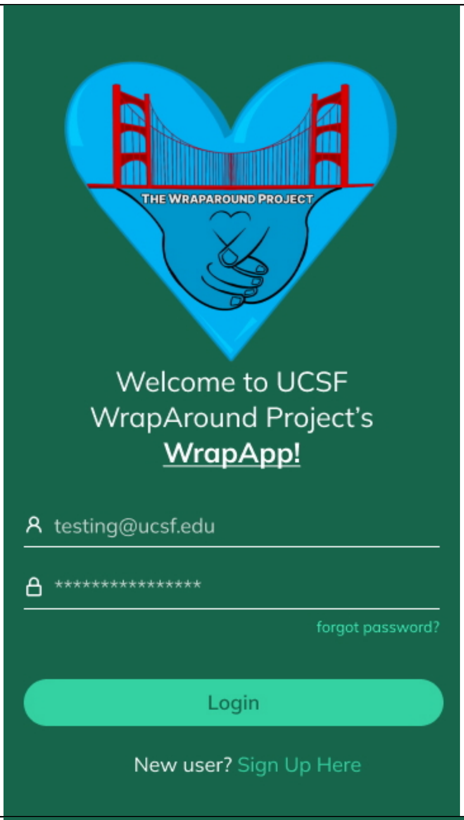
Client Dashboard	● Control over boundaries<br>● Intuitive and comprehensive design<br>● Standardization of verbiage and design choices	**Action:**<br>● Scroll through list of clients with most relevant information displayed in an organized and standard manner for consistent caseload tracking<br>● Add a new client with adjustable privacy settings on standardized and minimal input fields<br><br>**Features:**<br>● Client Dashboard: Client name, client avatar, client 'last check-in', client 'upcoming events', filter for clients, search bar, quick actions (call, text, view 'Care Plan')<br>● Add client button:	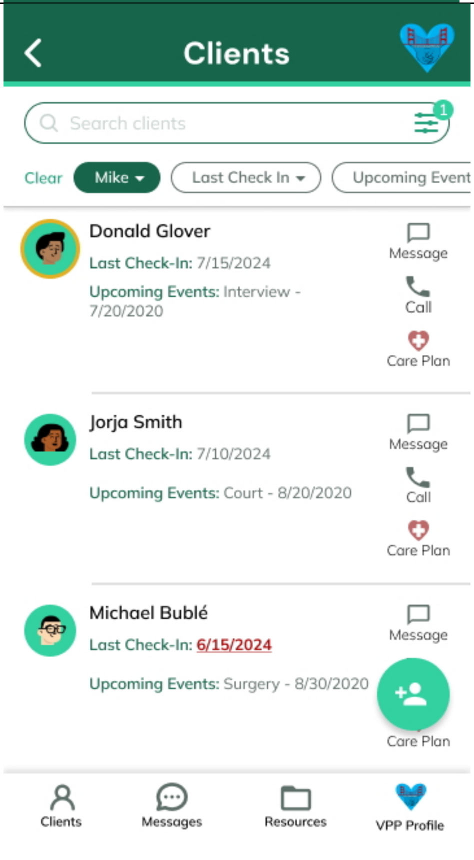

		Client name, client avatar, client name, client date of birth, client languages spoken, client nationality, client contact information, client neighborhood, client date of injury, client ‘disability status’, client notes, client ‘hospital entry status’, client ‘priority’, client safety concerns, client safety notes, client medical notes, client files, client crime report number, ‘Care Plan’ creation	
Client Profile	● Dynamic journey and sense of progress<br>● Personal connection<br>● Celebration of client successes<br>● Intuitive and comprehensive design<br>● Control over boundaries	**Action:**<br>● Review and edit details for a specific client that describe client’s whole story with adjustable privacy settings on standardized and minimal input fields<br>● Access and edit ‘Care Plan’ that includes milestones<br>● Access and edit achievements wireframe to celebrate client successes and build rapport with clients<br><br>**Features:**<br>● Client Profile: Client name, client avatar, client name, client date of birth, client languages spoken, client nationality, client contact information, client neighborhood, client date of injury,	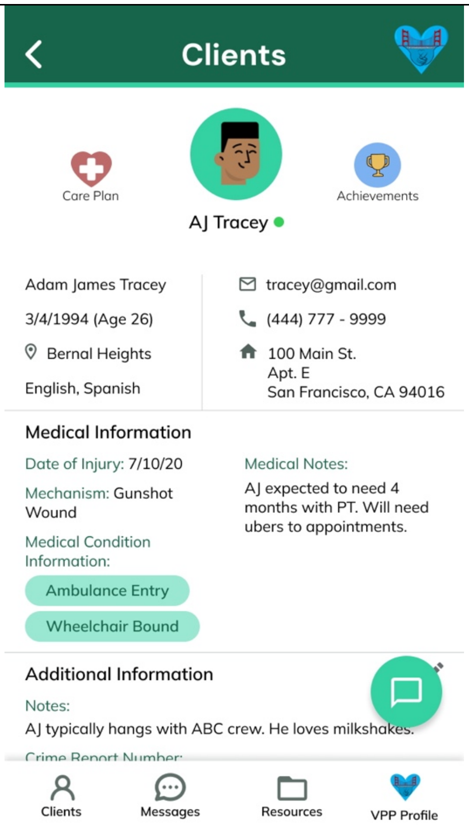

		client 'disability status', client notes, client 'hospital entry status', client 'priority', client safety concerns, client safety notes, client medical notes, client files, client crime report number<br>● Client achievements: view client badges, add client badges, customize client badges with personal message<br>● Care Plan: *see below*	
Care Plan	● Client empowerment<br>● Celebration of client successes<br>● Standardization of verbiage and design choices	**Action:**<br>● Monitor progress for client's personally maintained 'Care Plan' (built from template) that includes milestones, tasks, and subtasks for helping them change their life-course<br><br>**Features:**<br>● 'Care Plan': standard editable template, quick filters, navigation bar, milestones with progress bars, subtasks<br>● Subtasks: associated tasks, resources related to tasks, editing history	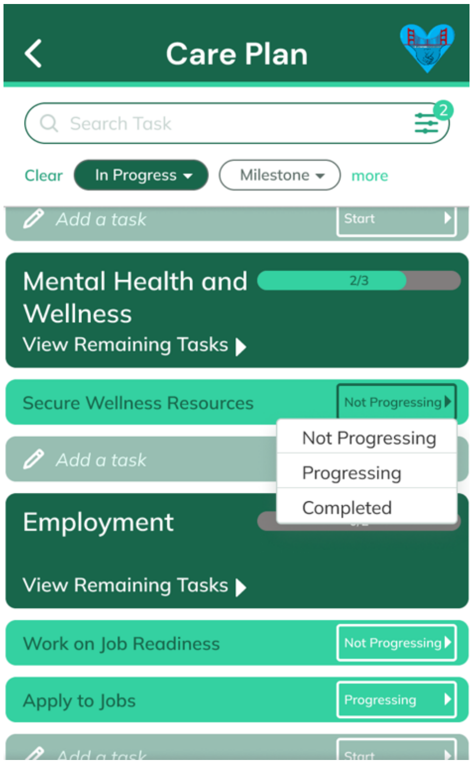

Resources	● Tailored resource curation<br>● Warm hand-off	**Action:**<br>● Review and scroll through standardized display of resources that are approved by VPPs<br>● Add/edit relevant resources to support clients<br>● Share resources to specific clients by associating to their 'Care Plan' tasks or sending over 'messaging' feature<br><br>**Features:**<br>● 'Employment' preview: jobs in list form, name of organization, application deadlines, date job was added, job requirements, ability to edit job, ability to send job to clients, filter for jobs, search for jobs<br>● Specific job: Name of organization, application deadlines, date job was added, job requirements, job location, how to apply to job, description of job, ability to edit resources, ability to send resource to clients<br>● Add/edit resource: Name of organization, application deadlines, date job was added, job requirements, job	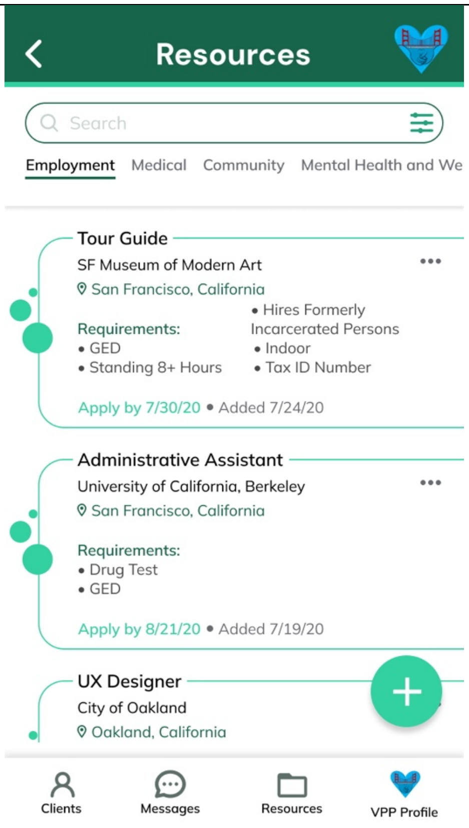

		location, how to apply to job, description of job<br>● Quick actions: edit resource, add resource to client's 'Care Plan', send resource to client on message	
Messaging	● Personal connection<br>● Control over boundaries	*Action*:<br>● Scroll and search message threads with clients<br>● Chat or call client through secure messaging feature<br>● Edit messaging settings<br><br>*Features*:<br>● Messaging: preview list of client messages, preview of client profile, alert of unopened messages, date of last message, time of last message, search bar<br>● Individual message thread: recipient field, send text option, send voice memo option, send resource option, quick action to call client or access Client Profile<br>● Message settings: editing 'Out of Office' automatic replies, setting options for client to receive help after-hours	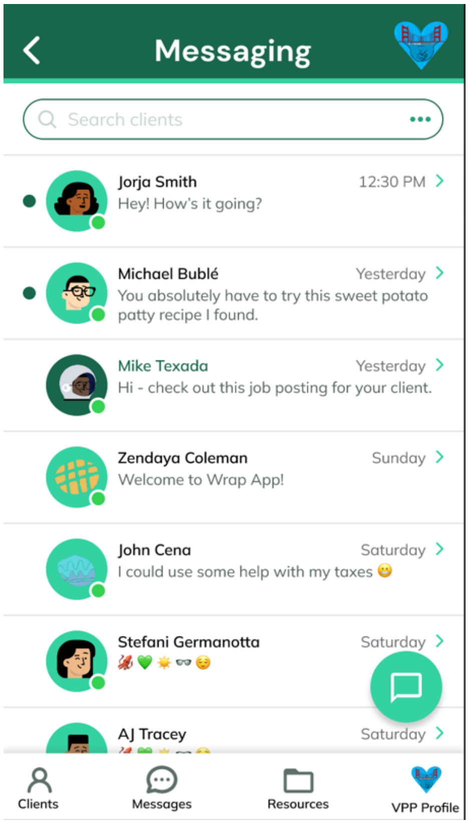

	● Control over boundaries<br>● Personal connection	**Action:**<br>● View/edit personal profile for VPP with privacy settings on standardized and minimal input fields<br>● View achievements which incorporates clients sharing gratitude for VPPs on their profiles<br><br>**Features:**<br>● VPP Profile: VPP Name, VPP contact information, VPP spoken languages, VPP biography, VPP interests, VPP favorite song, VPP favorite quote<br>● Achievements: view VPP badges received from clients	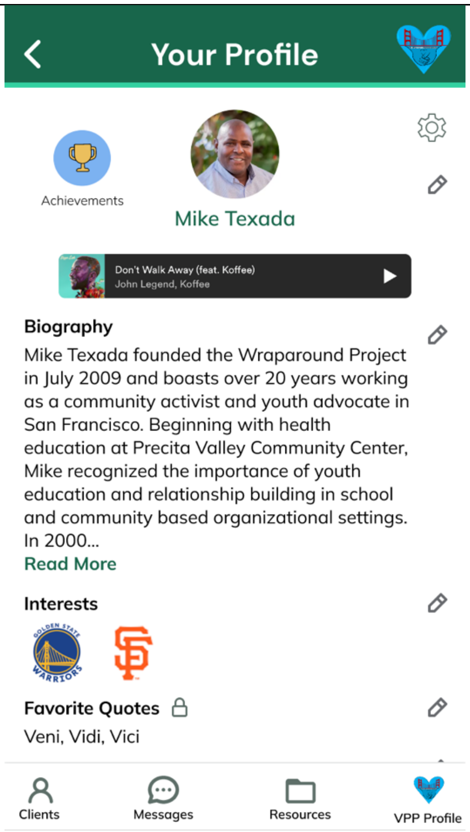
